# Editorial: Interdisciplinary surgical strategies for complex tumor defects in modern oncology

**DOI:** 10.3389/fonc.2023.1146719

**Published:** 2023-02-10

**Authors:** R. E. Horch, M. R. Kesting, S. Kersting, S. Fichtner-Feigl, A. Arkudas

**Affiliations:** ^1^ Department of Plastic and Hand Surgery, University Hospital Erlangen, Erlangen, Germany; ^2^ Department of Oral and Maxillofacial Surgery, University Hospital Erlangen, Erlangen, Germany; ^3^ Universitätsmedizin Greifswald, Greifswald, Germany; ^4^ Department of General and Visceral Surgery, University of Freiburg Medical Center, Freiburg, Germany

**Keywords:** cancer surgery, interdisciplinary surgical approaches, surgical tumor reconstruction, outcome, quality of life

Interdisciplinarity is a key element for modern cancer treatment. This is not only true for the interaction between various medical disciplines, but also a necessity due to the everlasting subspecialization within the field of surgery itself. Increasing surgical capabilities and technical advances within all specialized surgical disciplines have dramatically changed the face of modern surgical approaches to cure patients with malignant diseases (1).

While basically until the middle of the last century the sole removal of malignant tumors in any part of the human body was a challenge that had to be mastered by general surgeons on their own, it is now common sense and practice that specialized surgeons join the effort and come together to remove even advanced tumors and allow for safe simultaneous reconstructions (2–4). The various approaches depend on the extent of the tumor, infestation of vital structures, invovlement of neighbouring anatomic structures, presence of metastases, tissue conditions after neoadjuvant therapies etc.

Based on these newer concepts today even tumors which had hitherto been deemed to be unresectable or inoperable, may now be successfully operated upon. At the same time the collaboration of the resectional oncologic surgeon with reconstructive surgeons not only allows more radical tumor surgery but also can aid to reduce surgical complications (5) and enhances the remaining quality of life (QOL) for such patients (6, Peng et al.). Also, even when a tumor cannot be completely cured, interdisciplinary surgery can offer improve the quality of life in palliative situations (7). Pictures of decaying and unpleasant smelling tissue due to ruptured progressing tumors that hinder social contacts and lead to isolation hopefully belong to the past. This is another prospect of interdisciplinarity that yet has to be exploited whenever indicated.

Not only has the practice of surgery changed and undergone a significant evolution over the past 4 decades [e.g., introduction of new procedures and technological advancements (8, 9)], but newer specialties and subspecialties within surgery and surgical oncology have been created based on narrower anatomic regions and the application of increasingly advanced and specific technologies (10) (Zhang et al.; Gallina et al.). By their very nature, these growing surgical specialties and subspecialties tend to be consolidated in academic centers and larger urban regions where most teaching and training occurs (Lu et al.).

While on the one hand technical advances such as the evolution of minimally invasive surgery has been an important milestone in the field of surgical oncology or which has almost totally globally replaced open gastrectomy in treating gastric cancer, the individual knowledge of technically advanced instruments and tools, including high defintion imaging techniques is continuousy contributing to push the limits of possible resections and reconstructions forward over the course of the 20th and 21st century, based largely on the focus on specific organ systems or anatomic regions or specific surgical techniques (Cianci et al.). By integrating various surgical disciplines into tumor surgery more radical tumor resections can therefore be more safely performed and interdisciplinary reconstructions optimize the outcome of the individual patient`s treatment along with increased quality of life despite radical and oncologically sufficent cancer surgery.

The special issue comprises relevant hot topics and variants of interdisciplinary surgical oncology. Chen et al. describe their approach towards primary spinal Ewing sarcoma (ES)/peripheral primitive neuroectodermal tumors (pPNETs). This entities are extremely rare, and the current understanding of these tumors is poor. The authors aim to illustrate the clinical characteristics of primary spinal ES/pPNETs and to discuss prognostic factors by survival analysis. They show that otal en bloc resection can significantly improve PFS for primary spinal ES/pPNETs and adjuvant radiotherapy was a favorable factor for PFS in their patients. Total en bloc resection and adjuvant radiotherapy considerably improve overall survival (OS) for patients with primary spinal ES/pPNETs.

Thiele et al. compare the pros and cons of various perineal reconsturctive techniques following the resection of anorectal malignancies, which may result in extensive perineal/pelvic defects that require an interdisciplinary surgical approach involving reconstructive surgery. Their experience with either a myocutaneous gracilis flap (MGF) or a gluteal fold flap (GFF) compares the outcome regarding clinical key parameters. They conclude that MG-flaps and GF-flaps prove to be reliable and robust techniques for perineal/pelvic reconstruction. They suggest a decision-making based on distribution of adipose tissue for dead space obliteration, intraoperative patient positioning, and perforator vessel quality/distribution.

As a typical example of interdisciplinary oncologic surgery in this context the use of a transpelvic vertical rectus abdominis flap (VRAM) for relapsing or far advanced rectal and anal cancers in female patients with previous irradation prior to the surgical resection has been described in detail by Horch et al. This interdisciplinary approach can minimize the downside of abdomino-perineal resection or exenteration especially in women when parts of the vagina need to be resected. Derived from their experince with over 300 patients receiving pelvic and perineal reconstruction with a transpelvic vertical rectus abdominis myocutaneous (tpVRAM) flap they found that the tpVRAM flap is reliably perfused and helps to reduce long term wound healing desasters in the irradiated perineal/vaginal/gluteal region (**Figure 1**).

**Figure 1:**
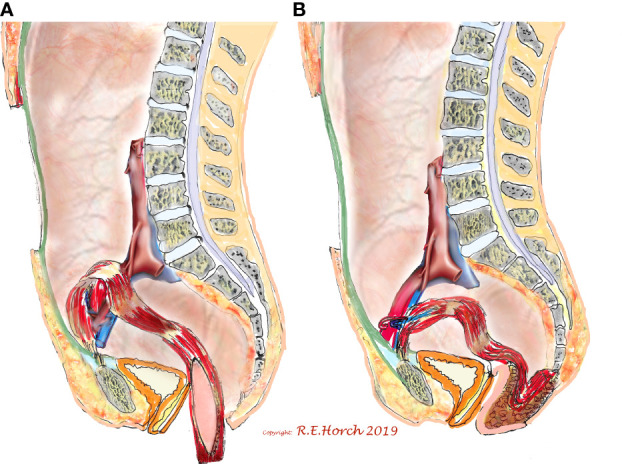
Schematic drawing of principle of vaginal wall reconstruction with pedicled transpelvic VRAM flap. **(A)** VRAM flap mobilized and routed through pelvis into resectional defect. **(B)** VRAM flap sutured to remaining anterior vaginal wall and constructing new posterior vaginal wall Horch et al.

Steiner et al. analyzed the interdisciplinary treatment of breast cancer which is based on the histological tumor type, the TNM classification, and the patient’s wishes. They demonstrate that following tumor resection and (neo-) adjuvant therapy strategies, breast reconstruction represents the final step in the individual interdisciplinary treatment plan. Their analysis comprises data from autologous microsurgical breast reconstruction with the deep inferior epigastric artery perforator (DIEP) or the muscle-sparing transverse rectus abdominis myocutaneous (ms-TRAM) flap. I a retrospective study focusing on the safety of autologous breast reconstruction upon mastectomy using abdominal free flaps in an academic university hospital they show a high success rate with comparatively few complications. Using preoperative computer tomography angiography, intraoperative fluorescence angiography, titanized hernia meshes for rectus sheath reconstruction, and venous coupler systems, autologous breast reconstruction with DIEP or ms-TRAM free flaps is a safe and standardized procedure in high-volume microsurgery centers.

Tan et al. studied the effectiveness and safety of the enhanced recovery after surgery (ERAS) protocol vs. traditional perioperative care programs for breast reconstruction. Ten studies were included in their meta-analysis. Their results suggest that ERAS protocols can decrease LOS and morphine equivalent dosing; therefore, they discuss that further larger, and better-quality studies that report on bleeding amount and patient satisfaction are needed to validate their findings.

Weitz et al. studied reconstructions of complex scalp after ablative resection or by post-traumatic tissue loss, that can make a simultaneous interdisciplinary two-team approach complicated, which is considered a major disadvantage regarding safety and operation time. Finally their data leed to the assumption that parascapular flap seem to be a good alternative for reconstruction of complex tumor defects of the scalp besides the latissimus dorsi flap. Stable long-term results and little donor site morbidity are enabled with good aesthetic outcomes and shorter operation time in an interdisciplinary two-team approach.

Cao et al. assessed the impact of enhanced recovery after surgery (ERAS) protocols in pancreaticoduodenectomy. They found no significant increase in mortality, readmission, reoperation, or delayed gastric emptying. Therefore they come to the conclusion that their analysis revealed that using ERAS protocols in pancreatic resections may help decrease the incidence of pancreatic fistula and infections. Furthermore, ERAS also reduces length of stay and cost of care. This study provides evidence for the benefit of ERAS protocols. Weber et al. describe that craniofacial osteosarcomas (COS) and extracranial osteosarcomas (EOS) show distinct clinical differences. They conclude that the reduced Gli1 expression in COS could be interpreted as reduced activation of the Hedgehog (Hh) signaling pathway. The increased M1 polarization and reduced Hh activation in COS could explain the low incidence of metastases in these osteosarcomas.

Zheng et al. aimed to compare survival outcome after receiving radiofrequency ablation (RFA) and surgical resection (SR) for solitary hepatocellular carcinoma (HCC) with size large as 5 cm. They found that by applying several effective sensitivity analyses, OS and CSS were similar between the patients with tumors smaller than 3 cm receiving RFA and SR. But SR may be a superior treatment option with better long-term outcome than RFA in patients with tumor measuring 3.1-5 cm.

Liang et al. performed a retrospective study to identify the prognostic significance of time to local recurrence (TLR) with regard to overall survival (OS) and survival after local recurrence (SAR) in patients with soft tissue sarcoma (STS) of the extremity and abdominothoracic wall. From their results they conclude that in patients with STS of the extremity and abdominothoracic wall, ELR after R0 resection indicated a worse prognosis than those with LLR, and TLR can be considered an independent prognostic factor for OS and SAR. Furthermore, local recurrence was significantly influenced by the depth and the histopathological grading of the primary tumor, and reoperation after local recurrence could improve survival, which means salvage surgery may still be the preferred treatment when there are surgical indications after recurrence.

## Conclusion

The contributions to this special issue highlight recent advances and approaches to the art of interdisciplinary oncological surgical and show how the challenges go along with functional organ or tissue preservation or restoration/reconstruction to maintain the highest possible QOL without reducing the aim of oncologic radicality.

## Author contributions

All authors listed have made a substantial, direct, and intellectual contribution to the work and approved it for publication.
